# Selenoprotein P as a Biomarker for Microvascular Complications in Type 2 Diabetes Mellitus: A Cross-Sectional Study in South India

**DOI:** 10.7759/cureus.103942

**Published:** 2026-02-19

**Authors:** Prabakaran Vaithinathan, Tutika Dynika, Sakthivel Vaithiyanathan, Ganesan C, Sridurga Mattyvanan

**Affiliations:** 1 Department of General Medicine, Vinayaka Mission's Medical College and Hospital, Vinayaka Mission's Research Foundation (Deemed to be University), Karaikal, IND; 2 Department of Internal Medicine, Vinayaka Mission's Medical College and Hospital, Vinayaka Mission's Research Foundation (Deemed to be University), Karaikal, IND; 3 Department of Biochemistry, Vinayaka Mission's Medical College and Hospital, Vinayaka Mission's Research Foundation (Deemed to be University), Karaikal, IND

**Keywords:** diabetic nephropathy, diabetic neuropathy, diabetic retinopathy, microvascular complications, selenoprotein p, type 2 diabetes mellitus

## Abstract

Background

Type 2 diabetes mellitus (T2DM) is a major global health concern, with microvascular complications, neuropathy, retinopathy, and nephropathy contributing substantially to morbidity. Selenoprotein P (SeP), a selenium-transporting antioxidant protein, has been implicated in metabolic dysregulation. This study examined the prevalence of microvascular complications in T2DM and explored their association with circulating SeP levels in a tertiary care center in South India.

Materials and methods

A cross-sectional study was conducted at Vinayaka Mission's Medical College, Karaikal (2022-2025), enrolling 80 adults with T2DM aged ≥40 years. Neuropathy was assessed using the 10 g monofilament test, retinopathy by fundus examination, and nephropathy by urine albumin-to-creatinine ratio (ACR). Serum SeP was quantified by enzyme-linked immunosorbent assay (ELISA). Analyses used SPSS version 26.0 (IBM Corp., Armonk, NY) with Pearson or Spearman correlation and independent t-test or Mann-Whitney U test, as appropriate.

Results

Among 80 participants (53.8% women; mean age: 58.84 ± 9.79 years), neuropathy was present in 45% (n = 36), retinopathy in 31.3% (n = 25), and nephropathy in 31.3% (n = 25). The mean SeP was 4.11 ± 1.47 µg/mL and tended to be higher with retinopathy (non-proliferative diabetic retinopathy {NPDR}: 4.52 ± 1.74; proliferative diabetic retinopathy {PDR}: 4.89 ± 1.26) than without (3.98 ± 1.34 µg/mL). Group differences were not statistically significant (retinopathy, p = 0.180; nephropathy, p = 0.187; and neuropathy, p = 0.120).

Conclusion

Microvascular complications are common in this T2DM cohort. Although not significant, higher SeP levels appeared to parallel retinopathy severity, supporting the further evaluation of SeP as a potential biomarker in larger or longitudinal studies.

## Introduction

Type 2 diabetes mellitus (T2DM) is a significant global health issue, characterized by insulin resistance or inadequate insulin secretion, resulting in chronic hyperglycemia [1]. The prevalence of T2DM in India is increasing due to urbanization, sedentary lifestyles, unhealthy eating habits, and genetic predispositions [2]. Microvascular complications, such as diabetic neuropathy, retinopathy, and nephropathy, impact up to 50% of individuals with T2DM, severely diminishing the quality of life, escalating healthcare expenses, and heightening mortality risk [3]. Finding dependable biomarkers for the early diagnosis and treatment of these issues is very important [4].

Selenoprotein P (SeP) is a selenium-transporting protein with several selenocysteine residues. It helps protect against oxidative stress and keep redox homeostasis by bringing selenium to tissues such as the brain, kidneys, and endocrine organs [5]. Physiological levels of SeP are protective; however, elevated concentrations are associated with insulin resistance and metabolic dysfunction, potentially worsening complications of T2DM [6]. Research presents contradictory results, with certain studies indicating elevated SeP levels in T2DM and others reporting decreases, highlighting the necessity for region-specific data [7].

Microvascular complications arise from oxidative stress and inflammation induced by hyperglycemia, leading to damage to small blood vessels [8]. Diabetic neuropathy, which affects both peripheral and autonomic nerves, causes numbness, tingling, or pain, which raises the risk of foot ulcers and amputations [9]. Diabetic retinopathy, a primary cause of blindness, advances from non-proliferative to proliferative stages as a result of retinal oxidative stress and neovascularization [10]. Diabetic nephropathy, marked by albuminuria and deteriorating renal function, constitutes a primary etiology of end-stage renal disease [11]. SeP's function in redox balance indicates that its dysregulation may lead to these complications, positioning it as a potential biomarker [12].

In South India, which has one of the highest rates of T2DM in the world, evidence on the relationship between SeP and microvascular complications remains limited. This research, undertaken at Vinayaka Mission's Medical College in Karaikal, Tamil Nadu, seeks to fill this void by assessing SeP levels in patients with T2DM exhibiting microvascular complications. The region's distinctive demographic and lifestyle characteristics, such as high-carbohydrate diets and restricted access to rural healthcare, require localized research [13]. Previous research has associated elevated SeP with metabolic parameters in T2DM; however, limited investigations have examined its specific correlation with microvascular outcomes [14].

This study seeks to evaluate the prevalence of microvascular complications in T2DM and examine SeP's viability as a biomarker. Identifying patients at risk early on could allow for timely interventions, such as better glycemic control or selenium modulation, to stop the disease from getting worse [5]. Furthermore, comprehending SeP's function may influence dietary recommendations, given that excessive selenium consumption has been linked to heightened diabetes risk [7]. This study enhances the literature by offering a thorough analysis within a South Indian cohort. We aimed to estimate the prevalence of microvascular complications in T2DM and examine their association with circulating SeP.

## Materials and methods

Study setting and participants

This hospital-based cross-sectional observational study was conducted at the Department of General Medicine, Vinayaka Mission's Medical College, Karaikal, Tamil Nadu, India, from June 2022 to May 2025. The hospital caters to a primarily rural demographic with a significant burden of T2DM, making it an optimal setting for the examination of diabetes complications. Adults aged ≥40 years with a confirmed diagnosis of type 2 diabetes mellitus of at least one-year duration, established according to American Diabetes Association diagnostic criteria (fasting plasma glucose, ≥126 mg/dL; postprandial plasma glucose, ≥200 mg/dL; or HbA1c, ≥6.5%), who attended inpatient or outpatient services during the study period and provided written informed consent were included [15,16].

Patients aged <40 years, those with gestational diabetes, individuals who had consumed selenium supplements within the preceding six months, patients with incomplete clinical records, and those unwilling to participate were excluded.

Sample size and sampling technique

The sample size was calculated using the single population proportion formula N = (Z² × P × Q) / L², as described by Charan and Biswas, where Z = 1.96 (95% confidence level), P = 0.75 (the estimated prevalence of microvascular complications), Q = 1-P (0.25), and L = 0.1 (10% margin of error), yielding 72 participants [15]. This was adjusted to 80 after adding 10% for potential nonresponders. Systematic random sampling was used to select participants from the hospital's T2DM registry, ensuring a representative cohort. A similar observational study in patients with diabetes and complications used a comparable sample size (44 with complications and 36 controls), supporting the adequacy of our planned sample size to detect clinically meaningful differences [16].

Study tools

A structured proforma gathered demographic information (age, sex, and address), clinical history (the duration of diabetes, treatment, and symptoms), and laboratory parameters. Automated analyzers were used to measure blood tests such as the complete blood count, renal function tests (urea and creatinine), lipid profiles (high-density lipoprotein {HDL}, low-density lipoprotein {LDL}, and triglycerides), and HbA1c. Researchers used an enzyme-linked immunosorbent assay (ELISA) to measure the levels of SeP. Diagnostic assessments included the 10 g Semmes-Weinstein monofilament test for the evaluation of diabetic peripheral neuropathy, and fundus examination for the classification of diabetic retinopathy into non-proliferative and proliferative stages, performed in accordance with recommendations from the American Diabetes Association [15]. Serum selenoprotein P levels were quantified using a validated ELISA method.

Study methodology

The participants underwent thorough clinical assessments. The monofilament test was used to check for neuropathy, and the loss of protective sensation (LOPS) was a sign of peripheral neuropathy. The examination of the fundus classified retinopathy into non-proliferative (non-proliferative diabetic retinopathy {NPDR}) or proliferative (proliferative diabetic retinopathy {PDR}) types. A diagnosis of nephropathy was made when the urine albumin-to-creatinine ratio (ACR) was greater than 30 mg/g, consistent with American Diabetes Association guidelines for chronic kidney disease risk stratification [15]. Five milliliters of blood were taken in Ethylenediaminetetraacetic acid (EDTA) and serum separator tubes, kept at -20°C, and tested for SeP using a validated ELISA kit. We kept an eye on glycemic control (fasting blood sugar {FBS}, postprandial blood sugar {PPBS}, and HbA1c), lipid profiles, and kidney function. Data were recorded systematically for accuracy.

Ethical considerations

The study protocol was reviewed and approved by the Vinayaka Mission's Medical College Institutional Ethical Committee, Karaikal (approval number: VMMC/2023/APR/04; dated 20/04/2023). Written informed consent was obtained from all the participants in their preferred language prior to enrolment. The confidentiality of participant data was strictly maintained, and participation was entirely voluntary.

Statistical analysis

Data were analyzed using SPSS version 26.0 (IBM Corp., Armonk, NY). Continuous variables, such as SeP levels and HbA1c, were expressed as means ± standard deviations (SD) or medians (IQR) based on data distribution assessed using the Shapiro-Wilk test. Categorical variables were expressed as frequencies and percentages. Pearson or Spearman correlation tests were used to evaluate associations between continuous variables. Independent samples t-tests or Mann-Whitney U-tests were applied for group comparisons as appropriate. A p-value of <0.05 was considered statistically significant.

## Results

Table 1 shows how old the participants who took part in the study. The largest group was 51-60 years old (36.2%, n = 29), which is the age group where T2DM is most common in India. Age group 61-70 years (27.5%, n = 22) was next, followed by people aged 50 years or younger (22.5%, n = 18) and people aged 71-80 years (12.5%, n = 10) and 81-90 years (1.3%, n = 1). This indicates that the study population predominantly comprised middle-aged to elderly individuals. These findings suggest that the study population reflects the typical demographic distribution of T2DM in South India.

**Table 1:** Age Distribution Among the Study Participants Age groups of the participants with type 2 diabetes mellitus (T2DM). Values are presented as n (%)

Age (Years)	n (%)
≤50	18 (22.5%)
51-60	29 (36.2%)
61-70	22 (27.5%)
71-80	10 (12.5%)
81-90	1 (1.3%)

Duration of diabetes

The average duration of diabetes in the study population was 8.36 ± 1.71 years. Most of the patients (36.2%, n = 29) had diabetes for 6-10 years, which suggests that the disease is chronic and may partly explain the high prevalence of complications observed. The patients with a duration exceeding 10 years (20.0%, n = 16) and those with 4-5 years (21.3%, n = 17) were notably substantial, whereas individuals with less than one year (8.8%, n = 7) and 1-3 years (13.7%, n = 11) were less prevalent, signifying a cohort characterized by considerable disease chronicity. Table 2 summarizes the duration of diabetes among the participants.

**Table 2:** Diabetes Duration Among the Study Participants Duration of T2DM among the participants. Values are presented as n (%) T2DM: type 2 diabetes mellitus

Duration of Diabetes (Years)	n (%)
<1 year	7 (8.8%)
1-3 years	11 (13.7%)
4-5 years	17 (21.3%)
6-10 years	29 (36.2%)
>10 years	16 (20.0%)

Prevalence of microvascular complications

Diabetic neuropathy was the most prevalent microvascular complication, present in 45.0% (36/80) of the participants, identified through the loss of protective sensation on the 10 g Semmes-Weinstein monofilament test. Retinopathy was detected in 31.3% (25/80) of the participants on fundus examination, and nephropathy was present in 31.3% (25/80) of the participants, defined by an albumin-to-creatinine ratio (ACR) of >30 mg/g (Table 3).

**Table 3:** Prevalence of Microvascular Complications Neuropathy was defined as the loss of protective sensation (LOPS) on 10 g Semmes-Weinstein monofilament testing; retinopathy was graded on fundus examination as NPDR or PDR; nephropathy was defined as a urine albumin-to-creatinine ratio (ACR) of >30 mg/g. Values are presented as n (%) NPDR, non-proliferative diabetic retinopathy; PDR, proliferative diabetic retinopathy

Complication	n (%)
Neuropathy (LOPS+)	36 (45%)
Retinopathy (NPDR + PDR)	25 (31.3%)
Nephropathy	25 (31.3%)

Selenoprotein P levels and retinopathy

Mean serum selenoprotein P (SeP) concentrations demonstrated a progressive increase with retinopathy severity. The participants without retinopathy had a mean SeP level of 3.98 ± 1.34 µg/mL, those with non-proliferative diabetic retinopathy (NPDR) had 4.52 ± 1.74 µg/mL, and those with proliferative diabetic retinopathy (PDR) exhibited the highest levels (4.89 ± 1.26 µg/mL). The comparison of SeP levels across retinopathy groups using one-way ANOVA showed no statistically significant difference (F = 1.756; p = 0.180), although the upward trend suggested a possible association between increasing SeP concentrations and retinopathy severity (Table 4).

**Table 4:** Mean Serum Selenoprotein P Levels by Retinopathy Status Comparison of serum selenoprotein P (SeP) levels across retinopathy groups using one-way ANOVA. Values are mean ± SD NPDR, non-proliferative diabetic retinopathy; PDR, proliferative diabetic retinopathy; SD, standard deviation

Retinopathy Status	SeP Levels (Mean ± SD, µg/mL)	F	P-value
Absent	3.98 ± 1.34	1.756	0.180
Non-proliferative (NPDR)	4.52 ± 1.74		
Proliferative (PDR)	4.89 ± 1.26		

Selenoprotein P levels and nephropathy

The participants with diabetic nephropathy (ACR > 30 mg/g) exhibited higher mean serum SeP levels (4.24 ± 1.48 µg/mL) compared to those without nephropathy (3.75 ± 1.42 µg/mL).

This difference was evaluated using an independent samples t-test and was not statistically significant (t = -1.332; p = 0.187). Although not statistically significant, the observed difference indicates a potential relationship between SeP concentration and renal dysfunction that warrants further exploration (Table 5).

**Table 5:** Mean Serum Selenoprotein P Levels by Nephropathy Status Comparison of serum selenoprotein P (SeP) levels between nephropathy groups using an independent samples t-test. Values are mean ± SD SD: standard deviation

Nephropathy Status	SeP Levels (Mean ± SD, µg/mL)	T-value	P-value
Present	4.24 ± 1.48	-1.332	0.187
Absent	3.75 ± 1.42		

## Discussion

This cross-sectional study conducted at Vinayaka Mission's Medical College, Karaikal, provides an in-depth analysis of microvascular complications in T2DM and their possible correlation with selenoprotein P (SeP) levels in a South Indian population. The high prevalence of complications, neuropathy (45%), retinopathy (31.3%), and nephropathy (31.3%), reflects the significant burden of T2DM in this region, consistent with global estimates suggesting that up to 50% of patients develop at least one microvascular complication [3].

The mean duration of diabetes was 8.36 ± 1.71 years, with 36.2% of the patients having diabetes for 6-10 years, indicating chronic hyperglycemia as a key contributor to microvascular damage. This aligns with evidence showing that long-term exposure to elevated blood glucose leads to endothelial injury and vascular dysfunction [8]. The mean HbA1c of 10.86% ± 2.01%, together with elevated FBS (173.90 ± 35.6 mg/dL) and PPBS (240.71 ± 52.3 mg/dL), confirms poor glycemic control, an established risk factor for the onset and progression of microvascular complications [9].

Neuropathy, detected in 45% of the participants using the 10 g monofilament test for the loss of protective sensation (LOPS), was the most frequent complication. This finding is in line with earlier studies reporting neuropathy in 30%-50% of the patients with T2DM, reaffirming its status as the most common microvascular manifestation [9]. Retinopathy and nephropathy, each present in 31.3% of the patients, correspond with global observations that these complications typically emerge after 5-10 years of disease duration [3]. The use of standardized diagnostic assessments, fundus examination for retinopathy and urine ACR for nephropathy, ensures reliable prevalence estimates and enhances the study's clinical relevance.

Mean SeP levels (4.11 ± 1.47 µg/mL) demonstrated a nonsignificant trend toward elevation in patients with retinopathy and nephropathy. Specifically, SeP levels increased with retinopathy severity: 3.98 ± 1.34 µg/mL in those without retinopathy, 4.52 ± 1.74 µg/mL in non-proliferative diabetic retinopathy (NPDR), and 4.89 ± 1.26 µg/mL in proliferative diabetic retinopathy (PDR). This progressive rise suggests that SeP may reflect oxidative stress and vascular injury associated with disease progression [10]. The lack of statistical significance may be attributed to the modest sample size (n = 80) or variability in SeP measurements, potentially influenced by dietary selenium intake, which varies considerably across India [7].

Similarly, patients with nephropathy (ACR > 30 mg/g) exhibited higher mean SeP levels (4.24 ± 1.48 µg/mL) compared to those without nephropathy (3.75 ± 1.42 µg/mL), corroborating findings from previous studies linking selenoproteins to renal dysfunction and albuminuria [11]. Although not statistically significant (p = 0.187), this pattern suggests that SeP may serve as an early indicator of renal microvascular involvement and warrants further validation in larger cohorts.

Discrepancies in SeP concentrations across studies have been reported. Misu et al. observed substantially higher SeP levels (e.g., 1032.4 ng/mL) in patients with T2DM compared to healthy controls, while our mean was 4.11 µg/mL [6]. Such differences may arise from methodological variations in assay calibration, differences in study populations, or disease stage. In our cohort, characterized by prolonged disease duration and poor glycemic control, SeP expression may differ from that seen in early-stage or better-controlled diabetes [14].

The upward trend of SeP levels in retinopathy is consistent with evidence implicating SeP in oxidative stress-mediated endothelial dysfunction and retinal neovascularization [10]. Likewise, elevated SeP levels among nephropathy patients align with findings by Roman et al., who associated selenoproteins with renal impairment [11]. Conversely, the absence of a significant association between SeP and neuropathy (p = 0.120) contrasts with the results of Herder et al., who reported increased SeP concentrations in diabetic neuropathy, possibly due to differences in neuropathy assessment methods or regional genetic variation [12].

The study population, with a mean age of 58.84 years and a predominance of individuals aged 51-60 years (36.2%), represents a mature diabetic cohort in whom complication rates tend to be higher [14]. The slight female predominance (53.8%) is consistent with regional epidemiological trends and may be influenced by factors such as obesity and hormonal variations [2].

A key limitation of this study is the absence of a nondiabetic control group, which restricts comparative interpretation of SeP levels against baseline values. Moreover, the cross-sectional design precludes causal inferences regarding SeP's role in the development or progression of complications. Dietary selenium intake, affected by geographic variations in soil selenium and local dietary habits, may also have influenced serum SeP levels, as excess selenium has been associated with insulin resistance [7]. This underscores the importance of region-specific studies integrating dietary and genetic data.

The strengths of this study include comprehensive clinical assessments, the use of standardized diagnostic tools, and a focus on a high-risk South Indian population. Nevertheless, the limited sample size may have reduced statistical power, and the lack of longitudinal follow-up restricted the evaluation of SeP as a predictive biomarker. The unavailability of data on dietary selenium intake and genetic polymorphisms influencing SeP expression also represents a limitation [5].

Overall, these findings highlight the need for stringent glycemic control and routine screening for microvascular complications in T2DM, particularly in high-prevalence regions such as South India. The observed elevation of SeP in retinopathy and nephropathy suggests its potential as a biomarker for early risk identification and stratification. Future studies incorporating dietary, genetic, and longitudinal data may further clarify SeP's role and inform targeted interventions such as antioxidant-based or selenium-modulating therapies [5,7].

Limitations

Study Design

Selenoprotein P (SeP) in diabetic microvascular problems cannot be causally or predictively interpreted due to the cross-sectional design.

Analysis and Sample

There was no multivariable correction for potential confounders, and the study was exploratory, using a small, single-center sample.

Measurement Considerations

Renal function and neuropathy evaluation techniques may have affected biomarker interpretation. SeP was assessed using a single ELISA approach.

## Conclusions

Microvascular complications remain highly prevalent among individuals with type 2 diabetes mellitus (T2DM), with neuropathy (45%), retinopathy (31.3%), and nephropathy (31.3%) emerging as the most frequent and clinically significant manifestations. Elevated selenoprotein P (SeP) levels showed a consistent trend toward association with retinopathy severity, suggesting its potential role as a biomarker for microvascular involvement. These findings emphasize the importance of strict glycemic control and regular screening for the early detection of complications. Further large-scale and longitudinal studies are warranted to validate the clinical utility of SeP and to elucidate its mechanistic role in diabetic microvascular pathology. Selenoprotein P is not a validated diagnostic or prognostic tool but rather an exploratory, correlational biomarker; the intent of its usage should be clear.

## References

[1] Reed J, Bain S, Kanamarlapudi V (2021). A review of current trends with type 2 diabetes epidemiology, aetiology, pathogenesis, treatments and future perspectives. Diabetes Metab Syndr Obes.

[2] Cade WT (2008). Diabetes-related microvascular and macrovascular diseases in the physical therapy setting. Phys Ther.

[3] Lam AA, Lepe A, Wild SH, Jackson C (2021). Diabetes comorbidities in low- and middle-income countries: an umbrella review. J Glob Health.

[4] Tommerdahl KL, Shapiro AL, Nehus EJ, Bjornstad P (2022). Early microvascular complications in type 1 and type 2 diabetes: recent developments and updates. Pediatr Nephrol.

[5] Gorini F, Vassalle C (2022). Selenium and selenoproteins at the intersection of type 2 diabetes and thyroid pathophysiology. Antioxidants (Basel).

[6] Misu H, Ishikura K, Kurita S (2012). Inverse correlation between serum levels of selenoprotein P and adiponectin in patients with type 2 diabetes. PLoS One.

[7] Rayman MP, Stranges S (2013). Epidemiology of selenium and type 2 diabetes: can we make sense of it?. Free Radic Biol Med.

[8] Cerf ME (2013). Beta cell dysfunction and insulin resistance. Front Endocrinol (Lausanne).

[9] Burgos-Morón E, Abad-Jiménez Z, Marañón AM (2019). Relationship between oxidative stress, ER stress, and inflammation in type 2 diabetes: the battle continues. J Clin Med.

[10] Demircan K, Jensen RC, Chillon TS (2023). Serum selenium, selenoprotein P, and glutathione peroxidase 3 during early and late pregnancy in association with gestational diabetes mellitus: prospective Odense Child Cohort. Am J Clin Nutr.

[11] Roman M, Lapolla A, Jitaru P (2010). Plasma selenoproteins concentrations in type 2 diabetes mellitus--a pilot study. Transl Res.

[12] Herder C, Saito Y, Spagnuolo MC (2024). Differential associations between selenoprotein P and distal sensorimotor polyneuropathy in people with and without diabetes: KORA F4/FF4 study. Free Radic Biol Med.

[13] Altinova AE, Iyidir OT, Ozkan C (2015). Selenoprotein P is not elevated in gestational diabetes mellitus. Gynecol Endocrinol.

[14] Demircan K, Chillon TS, Bang J, Gladyshev VN, Schomburg L (2024). Selenium, diabetes, and their intricate sex-specific relationship. Trends Endocrinol Metab.

[15] Charan J, Biswas T (2013). How to calculate sample size for different study designs in medical research?. Indian J Psychol Med.

[16] American Diabetes Association Professional Practice Committee (2024). Introduction and methodology: standards of care in diabetes-2024. Diabetes Care.

